# Knowledge, attitude and practices regarding vitamin D among adults in Ghana: a cross-sectional study

**DOI:** 10.1186/s12889-025-21370-x

**Published:** 2025-01-18

**Authors:** Abraham Ameyaw Kwabena, Benedicta Appiah, Samuel Ankomah Danso, Samuel Kwame Sopuruchi Agomuo, Samuel Kwarteng, Ebenezer Senu, Alfred Effah, Samuel Asamoah Sakyi, Linda Ahenkorah Fondjo

**Affiliations:** 1 Department of Medical Diagnostics, Faculty of Allied Health Sciences, Kwame Nkrumah University of Science and Technology, Kumasi, Ghana; 2 Department of Molecular Medicine, School of Medicine and Dentistry, Kwame Nkrumah University of Science and Technology, Kumasi, Ghana; 3 Department of Biological Sciences, School of Natural Sciences and Mathematics, The University of Texas at Dallas, Richardson, TX USA

**Keywords:** Vitamin D, Awareness, Knowledge, Attitudes, Practices

## Abstract

**Background:**

Vitamin D deficiency is a major public health concern, affecting approximately half of the world's population, partly due to limited public knowledge about vitamin D sources. However, there is lack of data on awareness, knowledge, attitudes, and practices regarding vitamin D in high-risk countries like Ghana. We investigated vitamin D awareness, knowledge and its associated factors in the Ghanaian population.

**Methods:**

This cross-sectional study involved 515 adults from Jaman South Municipal between January and June 2024. Questionnaires were used to obtain data on demographics, clinical characteristics, awareness and knowledge, attitude and practices towards vitamin D. Binary logistic regression analysis was used to determine the independent predictors of knowledge and practices regarding vitamin D. SPSS (version 26.0) was used for all statistical analysis. *P* < 0.05 was considered statistically significant.

**Results:**

Awareness, knowledge, attitude and practice level towards vitamin D was 61%, 56.9%, 63.7% and 73.2% respectively. Aged between 18–24 yrs [(aOR = 4.106, 95% CI: (1.523–11.072); *p* = 0.005)], being single [(aOR = 0.243, 95% CI: (0.065–0.904); *p* = 0.035)], having basic [(aOR = 0.216, 95% CI: (0.068–0.685); *p* = 0.009)] or secondary education [(aOR = 0.151, 95% CI: (0.073–0.313); *p* < 0.001)] and speaking English [(aOR = 3.553, 95% CI: (1.519–8.313); *p* = 0.003)] were the independent predictors of adequate knowledge on vitamin D. Having basic [(aOR = 9.058, 95% CI: (2.449–33.509); *p* = 0.001)] or secondary education [(aOR = 5.252, 95% CI: (2.508–10.996); *p* < 0.001)] increased the likelihood of good practices.

**Conclusion:**

There is high awareness but reduced knowledge on Vitamin D among the general public in Jaman South. Age, education, employment status, language were the factors associated with knowledge and practice regarding vitamin D. There is need for extensive health educational campaigns to the public to boost the knowledge levels on the importance of Vitamin D.

## Introduction

Vitamin D is a steroid hormone that is essential for the normal functioning of the body including the intestine, skin, bone, parathyroid glands, immune system, pancreas, and the healthy growth of a developing foetus [1]. Sunlight exposure is the primary source of vitamin D. Other sources include dietary supplements and foods like cereals and egg yolk [2]. Vitamin D exists in two forms: vitamin D3, and vitamin D2. The D3 form is produced by animals, including humans whereas vitamin D2 is produced by plants [3]. 25-Hydroxyvitamin D (25[OH]D), the active form of vitamin D, has a serum concentration cutoff of < 75 nmol/L, which is defined as vitamin D deficiency [4].

Vitamin D deficiency increases the risk of skeletal diseases such as rickets and osteoporosis, as well as non-skeletal diseases such as cardiovascular disease [5]. The lack of vitamin D leads to a number of medical problems that have been linked to higher death rates and negative outcomes, including immune system disorders, cardiovascular disease, type 2 diabetes obesity, metabolic syndrome, and various cancers [6]. Vitamin D stimulates cathelicidin and other defensins, which have immunological antimicrobial effects in the oral cavity [7]. In addition to regulating calcium levels, vitamin D is essential for craniofacial development and maintaining dental health [8].

The global prevalence of vitamin D deficiency is a significant public health concern, affecting approximately 15.7% of the global population [9]. A study, including systemic reviews and meta-analyses found that poor vitamin D status is prevalent in over 30% of Africans, and over 18% were severe cases with Vitamin D levels below 30nmol/L [10]. Despite Ghana's abundant sunshine, vitamin D deficiency is prevalent among the adult population, with reported rates ranging from 43.6% to 81.7% across various age groups [1, 11, 12]. The incidence in the middle belt, which includes Kumasi, was roughly 45.3%, and it was linked to a decrease in vitamin D intake and lack of knowledge on the vitamin [1].

A recent international report on vitamin D deficiency in the developing world recommended improvement in the knowledge and awareness about vitamin D and its impact of health outcomes among adult population [13]. Research indicates that inadequate knowledge, poor attitudes and practices regarding vitamin D, its sources and benefits contributes to the prevalence of deficiency [14]. Despite the proven importance of vitamin D for general health and the great threat vitamin D deficiency poses, there is still no study that has examined the knowledge, awareness, attitudes and practices regarding vitamin D among adults in Ghana, following the high prevalent rates. By investigating these factors, specific gaps could be identified to warrant tailored interventions to reduce vitamin D deficiency prevalence. Thus, this study aimed to assess the knowledge, awareness, attitudes, and practices concerning Vitamin D among adults in the Jaman South Municipality of Ghana.

## Materials and methods

### Study design

This study employed a cross-sectional survey via questionnaire study design to evaluate the knowledge, attitudes, awareness and practices regarding Vitamin D among the general adult population at the Jaman South Municipal, Ghana between January and June 2024.

### Study site

The study was conducted in Jaman South Municipality, Bono Region, Ghana, with approximately 53% of its residents being adults aged 18 and over [15]. The study specifically took place at St. Mary’s Catholic Hospital, which is a municipal hospital serving over 40 communities. The hospital serves as a referral hospital to all the rural clinics and Community-Based Health Planning and Services (CHPS) Compounds in the Jaman South district, making it an ideal site to capture a diverse sample of adults from both rural and peri-urban backgrounds. The location of the hospital also allows for a comprehensive assessment of knowledge, attitudes, and practices regarding Vitamin D across varied sociodemographic groups.

### Study population and study size estimation

The study was conducted among adults residing in the Jaman South District. The sample size for this study was determined using the Taro Yamane formula;

n = $\frac{\text{N}}{1+\text{ N}(\text{e})2}$, n is the required sample size from the population under study, N is the whole population that is under study (57,962) [15] and e as the sampling error (0.05).

$$\text{n }=\frac{57962}{ 1+\text{ 57,962}(0.05)2},\text{n }=397.26$$

The minimum sample size required for the study was 397 based on a target population size of 57,962 and a sampling error of 0.05. However, a total of 515 study participants were recruited to increase statistical power. At the Outpatient Department (OPD), patients who met the inclusion criteria were recruited using simple random sampling. Patients were asked to pick a paper card from a container containing cards marked with either "0" or "1." Those who picked a card marked with "1" were included in the study, while those who picked "0" were excluded.

### Inclusion and exclusion criteria

The study included adults aged 18 years and older living in the specified study area who consented to participate in the study, while excluding those who met the age and residency requirements but were unwilling to participate, as well as individuals under 18 years of age.

### Data collection

A well-structured questionnaire was used to obtain data on participants’ sociodemographic and clinical characteristics, knowledge, attitudes, and practices towards vitamin D. The questionnaire was adopted and modified based on previous studies consisting of validated items across a range of dimensions [1, 16–18]. The questionnaire consisted of four sections: Part A included sociodemographic and clinical details such as age, ethnicity, gender, education, income, occupation, medical/surgical history, and lifestyle habits. Part B assessed awareness and knowledge about vitamin D, including whether participants had heard of it and what sources of information they had about its benefits and sources. Part C examined attitudes toward vitamin D and sun exposure based on agreement with various statements. Section D focused on vitamin D consumption, daily intake, and sun exposure in participants' daily lives.

### Measurements

A single question (Question 18) was used to determine awareness: "Yes" for aware and "No" for unaware. Knowledge was evaluated through eight questions (Questions 20–27), scoring up to 26 points, with scores ≥ 13 indicating adequate knowledge and < 13 indicating inadequate knowledge. Attitude was assessed using five questions (Questions 28–32) on a scale where "Strongly disagree" scored 0, "Disagree" scored 1, "Neutral" scored 2, "Agree" scored 3, and "Strongly agree" scored 4, allowing for a maximum score of 20 points. Scores ≥ 10 indicated a satisfactory attitude, while < 10 indicated an unsatisfactory attitude. For practices, nine questions (Questions 33–38) were scored, with a maximum of 9 points, where scores > 4.5 indicated good practices and < 4.5 indicated bad practices.

### Data analysis

Data obtained was entered and cleaned in Microsoft Excel 2021 and analyzed using SPSS version 26. Categorical variables were summarized as frequencies and percentages. The binary logistic regression analysis model was used to determine the independent predictors of awareness, knowledge, attitudes and good practices regarding vitamin D. *P* < 0.05 was considered statistically significant.

## Results

### Sociodemographic characteristics of the study participants

Of the 515 study participants, majority were females (53.8%) and were within 18–24 years (45.6%). Most of the participants were single (56.9%) and had attained tertiary education (38.4%). Considering employment status, 45.4% were self-employed, 43.5% students, 6.2% unemployed, 3.9% formally employed, and 1% retired. Moreover, majority (69.9%) earned below GH₵ 500 (Table 1).

**Table 1:** Sociodemographic characteristics of the general adult in Jaman South

Variables	Frequency (*n* = 515)	Percentage (%)
**Age (yrs)**		
18–24	235	45.6
25–39	146	28.3
≥ 40	134	26.0
**Gender**		
Female	277	53.8
Male	238	46.2
**Marital Status**		
Single	293	56.9
Married	183	35.5
Divorced	9	1.7
Widow(er)	30	5.8
**Highest Educational Level**		
Non-formal Education	3	0.6
Basic Education	168	32.6
SHS	146	28.3
Tertiary	198	38.4
**Employment status**		
Unemployed	32	6.2
Student	224	43.5
Self-employed	234	45.4
Formal	20	3.9
Retired	5	1.0
**Monthly income (GH₵)**		
< 500	360	69.9
500–1000	127	24.7
1000–2000	16	3.1
> 20,000	12	2.3
**Religion**		
Christianity	483	93.8
Muslim	31	6.0
Others	1	0.2
**Language**		
Twi	499	96.9
English	327	63.5
Others	90	17.5
**BMI (Kg/m**^**2**^**)**		
Normal	458	88.9
Underweight	10	1.9
Overweight	46	8.9
Obese	1	0.2

Data presented as frequencies and percentages (%)

*SHS* Senior Secondary School, *BMI* Body Mass index

#### Clinical and lifestyle characteristics of the general adults in Jaman South

Few of the study participants had an active health condition (6.4%) with hypertension being the most prevalent (33.3%), followed by bone disease (21.2%), asthma (6.1%), and diabetes (6.1%). Regarding lifestyle habits, majority (99.2%) were non-smokers (99.2%), with 8.7% known to consume alcoholic beverages. Nearly half (48.2%) of the participants engage in outdoor games sometimes, while (18.1%) participate always, and (33.8%) never engage in outdoor games (Table 2).

**Table 2:** Clinical and lifestyle characteristics of study participants

Variable	Frequency (*n* = 515)	Percentage (%)
**Any kind of health condition?**		
Yes	33	6.4
No	482	93.6
**Health conditions**		
Asthma	2	6.1
Diabetes	2	6.1
Bone diseases	7	21.2
Fever	1	3.0
HBP	11	33.3
Headache	2	6.1
Hernia	1	3.0
Infertility	1	3.0
Prostate cancer	1	3.0
Skin rash	1	3.0
Stroke	2	6.1
Ulcer	2	6.1
**Smoking**		
Yes	4	0.8
No	511	99.2
**Consumption of alcoholic beverages**		
Yes	45	8.7
No	470	91.3
**Engage in outdoor games**		
Always	93	18.1
Sometimes	248	48.2
Never	174	33.8

Data presented as frequencies and percentages (%)

*HBP* High Blood Pressure

### Awareness and knowledge responses regarding vitamin D

**Fig. 1:**
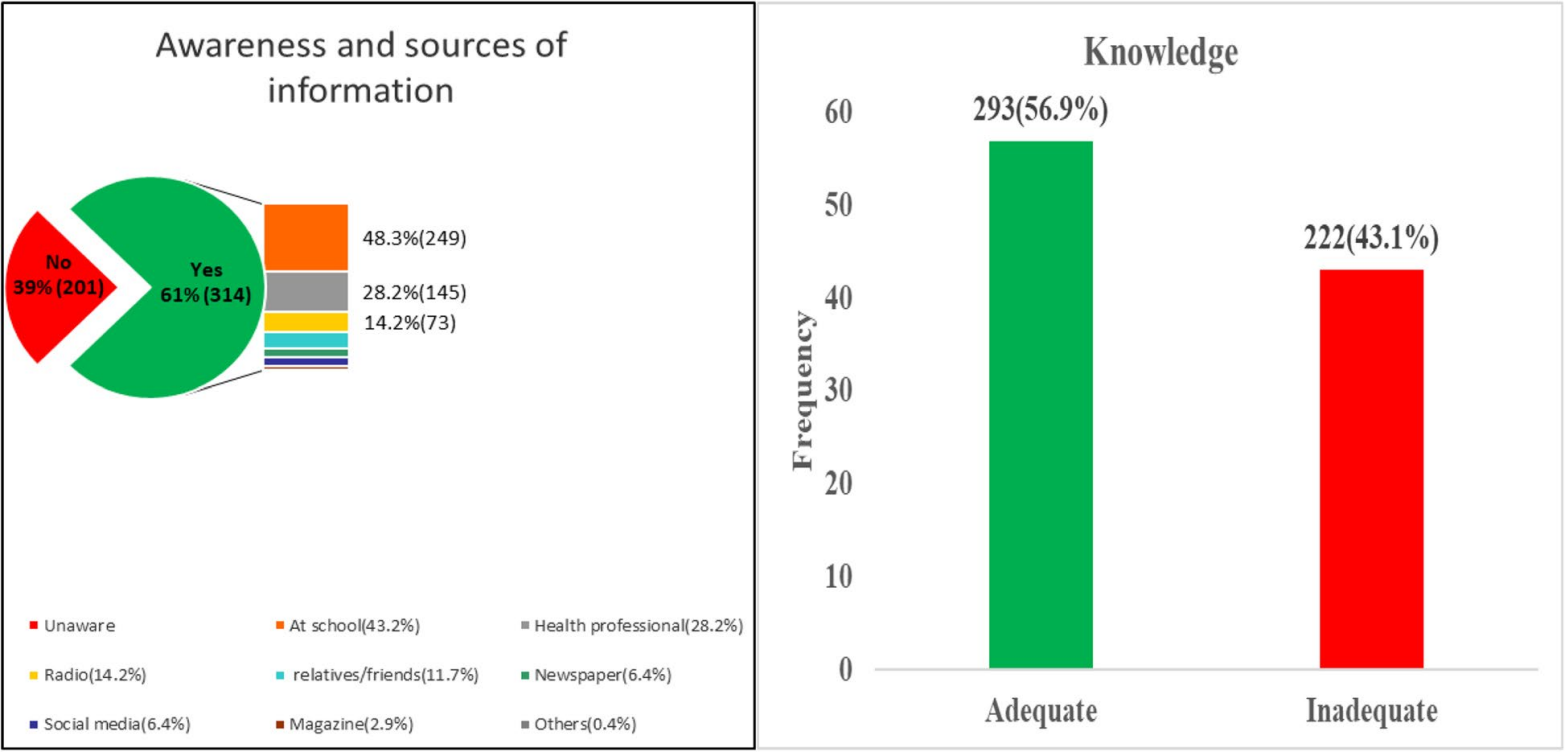
Awareness, source of information on vitamin D and knowledge level on vitamin D

Majority (61%) of the study participants were aware of vitamin D and reported receiving information about vitamin D, primarily from school (48.3%) and health professionals (28.2%) (Fig. 1). The importance of vitamin D for general health was acknowledged by 67% of participants, with 62.5% recognizing its benefits for bone health. Regarding sources of vitamin D, 53% identified sun exposure, followed by diet (42.7%), and supplements (21.2%). Vegetables and Fruits (32.8%) Fatty fish (24.9%), milk (22.7%), and eggs (30.5%) were recognized as good dietary sources. However, about 43% were unaware of the dietary sources. Participants' knowledge on the relevance vitamin D to various health conditions was varied, with 58.4% linking it to bone health, 20% to cancer, and smaller percentages to diabetes (6.4%), heart diseases (7.4%), and pregnancy (8.9%). About 41% of participants believed sun exposure is harmful to the skin, while 86.6% agreed that outdoor games and activities are beneficial for sun exposure. Finally, 66.8% knew that vitamin D status can be checked in health laboratories, highlighting a need for further education on this topic (Table 3).

**Table 3:** Knowledge responses regarding vitamin D among study participants

Variable	Frequency (*n* = 515)	Percentage (%)
**Effect of Vitamin D on general health is important**		67.0
Yes	345	67.0
No	170	33.0
**Vitamin D is good for bone health**		
True	322	62.5
False	193	37.5
**According to you, Vitamin D is relevant in which of the following health conditions?**		
Bone health	301	58.4
Cancer	103	20.0
Diabetes	33	6.4
Heart diseases	38	7.4
Pregnancy	46	8.9
Health effect	194	37.7
**Where do you think the body gets vitamin D from?**		
Diet	220	42.7
Sun exposure	273	53.0
Supplement	109	21.2
Exercise	18	3.5
Air	6	1.2
Water	7	1.4
Don't know	188	36.5
**Good dietary sources of vitamin D**		
** Vegetables and fruits**	169	32.8
Milk	117	22.7
Fatty fish (Salmon, Sardines)	128	24.9
Olive oil	43	8.3
Egg	157	30.5
Chicken	50	9.7
Red meat	54	10.4
Don’t know	220	42.7
**Is exposure to sunlight harmful for the skin?**		
Yes	211	41.0
No	304	59.0
**Outdoor games /activities are good for exposure to the sun**		
True	446	86.6
False	69	13.4
**Vitamin D status can be checked in the health laboratories**		
Yes	344	66.8
No	32	6.2
Don’t know	139	27.0

Variables are presented as frequencies and percentages

### Attitude responses regarding vitamin D amongst adults in Jaman South

Out of 515 participants in the study, most participants (44.5%) like to expose themselves to sunlight all the time, whiles 27.4% disagree with this statement. In contrast, 32% often use protective measures such as parasols and sunscreen lotion to prevent sunshine. Concern about current vitamin D levels is evident, with over half (51.3%) of participants expressing concern, while a smaller percentage (9.9%) agree that they are concerned. The willingness to undergo testing for vitamin D is high, with a significant majority (68.9%) strongly agreeing to undergo testing if a medical condition demands it. Regarding vitamin D supplements, a majority (55.9%) of participants are willing to take them, while a smaller percentage (11.8%) agree to take supplements (Table 4).

**Table 4:** Attitude responses regarding vitamin D among study participants

Variable	Frequency (%)				
	**strongly disagree**	**Disagree**	**Neutral**	**Agree**	**Strongly agree**
**I like to expose to sunlight all the time**	229(44.5)	141(27.4)	48(9.3)	67(13.0)	30(5.8)
**I often use a parasol (sunshade or umbrella) and sunscreen lotion to prevent sunshine**	134(26)	112(21.7)	57(11.1)	165(32)	47(9.1)
**I am concerned about my current vitamin D levels**	264(51.3)	145(28.2)	45(8.7)	51(9.9)	10(1.9)
**I am always willing to undergo test for vitamin D if a medical condition demands it**	15(2.9)	10(1.9)	14(2.7)	121(23.5)	355(68.9)
**I am always willing to take vitamin D supplements**	288(55.9)	115(22.3)	28(5.4)	61(11.8)	23(4.5)

Categorical variables were presented as frequencies and percentages

### Attitude level on vitamin D among study participants

**Fig. 2:**
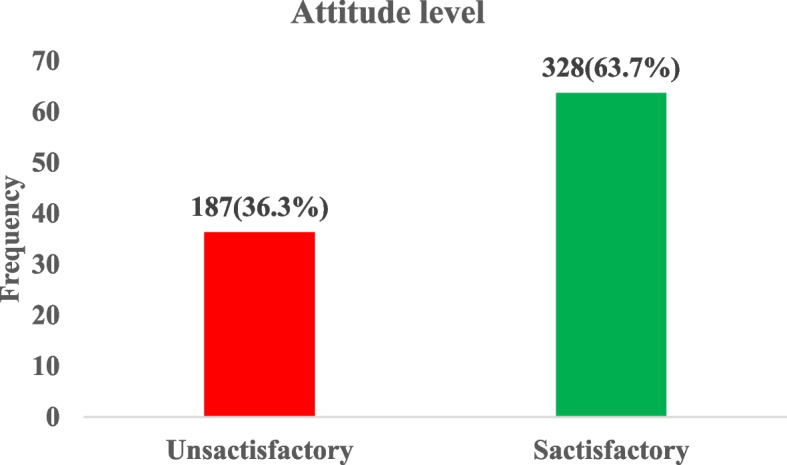
Attitude level regarding vitamin D among study participants

Figure 2 shows that 63.7% of the participants shows satisfactory attitude towards vitamin D with lesser percentage (36.3%) having unsatisfactory attitude.

### Practices responses regarding vitamin D among study participants

Only 5.2% of the study participants took vitamin D supplements, while the majority (97.5%) purchased vitamin D-fortified foods. Regarding sun protection, the majority (91.7%) do not use SPF-containing creams. The average daily sun exposure varies, with nearly half (43.7%) spending 30–60 min outdoors, 31.5% spending more than 60 min, and about 24.8% spending less than 30 min in sunlight daily. Majority (69.5%) of the participants walk outdoors daily for sufficient sunlight exposure. Only about 2% have undergone vitamin D testing (Table 5).

**Table 5:** Practices responses regarding vitamin D amongst adult in Jaman South

Variable	Frequency(*n* = 515)	Percentage (%)
**Ever taken vitamin D supplements**		
Yes	27	5.2
No	488	94.8
**Purchase vitamin D foods**		
Yes	502	97.5
No	13	2.5
**Not using Sun Protective Factor (SPF) contain creams**		
True	472	91.7
False	43	8.3
**Average length of daily sun exposure**		
< 15 min	16	3.1
51-30 min	112	21.7
30–60 min	225	43.7
> 60 min	162	31.5
**Walk outdoor daily for sufficient sunlight exposure**		
Yes	358	69.5
No	157	30.5
**Ever undergone vitamin D testing**		
Yes	10	1.9
No	505	98.1

Data were presented as frequencies and percentages

### Level of good practices regarding vitamin D among adult in Jaman South

**Fig. 3:**
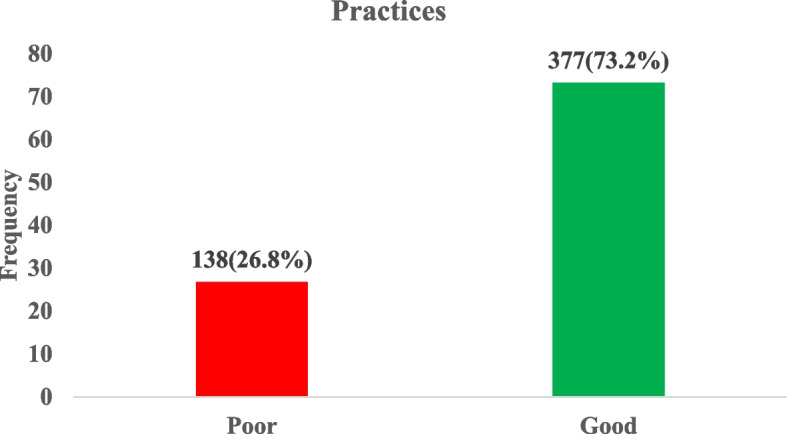
Level practices regarding vitamin D among study participants

Nearly three quarters of the study participants had good practice regarding Vitamin D (73.2%) while 26.8% had poor practice (Fig. 3).

### Sociodemographic and lifestyle predictors of adequate knowledge about vitamin D

In univariate analysis, individuals aged 18–24 (cOR: 7.00, 95% CI: 4.37–11.23; *p* < 0.001) and 25–39 (cOR: 2.13, 95% CI: 1.31–3.47; *p* = 0.002) were more likely to have adequate vitamin D knowledge compared to those above 40 years. Being single (cOR: 5.35, 95% CI: 2.36–12.14; *p* < 0.001), a student (cOR: 18.78, 95% CI: 7.27–48.50; *p* < 0.001), and English-speaking (cOR: 7.65, 95% CI: 5.10–11.47; *p* < 0.001) were associated with increased likelihood of adequate knowledge, while no formal education (cOR: 0.07, 95% CI: 0.01–0.75; *p* = 0.029), basic (cOR: 0.05, 95% CI: 0.03–0.09; *p* < 0.001), and SHS education (cOR: 0.12, 95% CI: 0.07–0.20; *p* < 0.001) were associated with lower chances of having adequate knowledge.

However, after adjusting for putative confounders in a multivariate analysis, age 18–24 (aOR: 4.11, 95% CI: 1.52–11.07; *p* = 0.005), being single (aOR: 0.24, 95% CI: 0.07–0.90; *p* = 0.035), basic (aOR: 0.22, 95% CI: 0.07–0.69; *p* = 0.009) and SHS education (aOR: 0.15, 95% CI: 0.07–0.31; *p* < 0.001), being a student (aOR: 5.38, 95% CI: 1.68–17.15; *p* = 0.005), and speaking English (aOR: 3.55, 95% CI: 1.52–8.31; *p* = 0.003) were the independent predictors of adequate knowledge (Table 6).

**Table 6:** Sociodemographic and lifestyle predictors of adequate knowledge of Vitamin D

Variable	Adequate knowledge (*n* = 293)	cOR (95% CI)	*p*-value	aOR (95% CI)	*p*-value
**Age (yrs)**					
18–24	179 (61.1)	7.00 (4.37–11.23)	**< 0.001**	4.11 (1.52–11.07)	**0.005**
25–39	72 (24.6)	2.13 (1.31–3.47)	**0.002**	1.83 (0.90–3.73)	0.096
≥ 40	42 (14.3)	1		1	
**Marital Status**					
Single	204 (69.6)	5.35 (2.36–12.14)	**< 0.001**	0.24 (0.07–0.90)	**0.035**
Married	77 (26.3)	1.70 (0.74–3.90)	0.215	-	-
Divorced	3 (1.0)	1.17 (0.24–5.73)	0.849	-	-
Widow(er)	9 (3.1)	1		1	
**Highest Educational Level**					
Non-formal Education	1 (0.3)	0.07 (0.01–0.75)	**0.029**	0.27 (0.01–5.11)	0.380
Basic Education	49 (16.7)	0.05 (0.03–0.09)	** < 0.001**	0.22 (0.07–0.69)	**0.009**
SHS	68 (23.3)	0.12 (0.07–0.20)	** < 0.001**	0.15 (0.07–0.31)	** < 0.001**
Tertiary	175 (59.7)	1		1	
**Employment status**					
Unemployed	6 (2.0)	1		1	
Student	182 (62.1)	18.78 (7.27–48.50)	** < 0.001**	5.38 (1.68–17.15)	**0.005**
Self-employed	83 (28.3)	2.38 (0.94–6.02)	0.067	-	-
Formal	19 (6.5)	82.33 (9.14–741.65)	** < 0.001**	43.75 (3.29–581.86)	**0.004**
Retired	3 (1.0)	6.50 (0.88–47.90)	0.066	-	-
**Religion**					
Christian	265 (90.4)	1		1	
Muslim	28 (9.6)	7.68 (2.30–25.60)	**0.001**	1.96 (0.50–7.73)	0.338
Others	0 (0.0)	-		-	-
**Language**					
English					
Yes	242 (82.6)	7.65 (5.10–11.47)	** < 0.001**	3.55 (1.52–8.31)	**0.003**
No	51 (17.4)	1		1	
Others					
Yes	71 (24.2)	3.42 (1.99–5.87)	** < 0.001**	1.78 (0.89–3.55)	0.102
No	222 (75.8)	1		1	
**Do you smoke?**					
Yes	0 (0.0)	1		1	
No	293 (100.0)	-		-	-
**Consumption of alcoholic beverages**					
Yes	18 (6.1)	1		1	
No	275 (93.9)	2.12 (1.13–3.95)	**0.019**	1.16 (0.49–2.73)	0.741
**Engage in outdoor games**					
Always	58 (19.8)	1.82 (1.09–3.04)	**0.023**	0.82 (0.38–1.75)	0.603
Sometimes	152 (51.9)	1.74 (1.17–2.57)	**0.006**	1.01 (0.57–1.77)	0.985
Never	83 (28.3)	1		1	
**Monthly income**					
< 500	207 (70.6)	0.12 (0.02–0.96)	**0.046**	0.38 (0.03–5.73)	0.484
500–1000	65 (22.2)	0.10 (0.01–0.76)	**0.027**	0.30 (0.02–4.50)	0.384
1000–2000	10 (3.4)	0.15 (0.02–1.49)	0.105	-	-
> 2000	11 (3.8)	1		1	

*cOR* crude odds ratio, *aOR* adjusted odds ratio, *CI* Confidence interval, *p* < 0.05 and bolded means statistically significant

### Sociodemographic and lifestyle predictors of satisfactory attitude level towards vitamin D

In the univariate analysis, being male (cOR = 3.99, 95% CI: 2.69–5.92; *p* < 0.001) and sometimes engaging in outdoor games (cOR = 1.88, 95% CI: 1.26–2.81; *p* = 0.002) significantly increased the likelihood of a satisfactory attitude toward vitamin D. Not consuming alcohol (cOR = 0.41, 95% CI: 0.19–0.87; *p* = 0.020) significantly decreased this likelihood.

However, after adjusting for potential confounders in a multivariate logistics regression analysis model, being male (aOR = 3.64, 95% CI: 2.41–5.50; *p* < 0.001) and sometimes engaging in outdoor games (aOR = 1.67, 95% CI: 1.08–2.56; *p* = 0.020) were associated with increased likelihood of satisfactory attitude towards vitamin D (Table 7).

**Table 7:** Sociodemographic and lifestyle predictors of satisfactory attitude level towards vitamin D

Variable	Satisfactory Attitude (*n* = 314)	cOR (95% CI)	*p*-value	aOR (95% CI)	*p*-value
**Gender**					
Female	138 (42.1)	1		1	
Male	190 (57.9)	3.99 (2.69–5.92)	** < 0.001**	3.64 (2.41–5.50)	** < 0.001**
**Monthly income**					
< 500	259 (68.7)	0.13 (0.02–1.04)	0.055	0.35 (0.04–2.86)	0.324
500–1000	97 (25.7)	0.25 (0.03–2.00)	0.191	0.51 (0.06–4.33)	0.538
1000–2000	14 (3.7)	0.15 (0.02–1.49)	0.105	0.24 (0.02–2.54)	0.238
> 2000	7 (1.9)	1		1	
**Twi**					
Yes	369 (97.9)	0.02 (0.05–1.08)	0.063	0.34 (0.07–1.66)	0.182
No	8 (2.1)	1		1	
**Consumption of alcoholic beverages**					
Yes	36 (11.0)	1		1	
No	292 (89.0)	0.41 (0.19–0.87)	**0.02**	0.55 (0.25–1.22)	0.139
**Engage in outdoor games**					
Always	61 (18.6)	1.59 (0.94–2.67)	0.083	1.08 (0.61–1.91)	0.787
Sometimes	172 (52.4)	1.88 (1.26–2.81)	**0.002**	1.67 (1.08–2.56)	**0.02**
Never	95 (29.0)	1		1	

*cOR* crude odds ratio, *aOR* adjusted odds ratio, *CI* Confidence interval, *p* < 0.05 and bolded means statistically significant

### Sociodemographic and lifestyle predictors of good practice regarding vitamin D

In the univariate logistic regression analysis, compared to those aged 40 and above, individuals aged 18–24 had a significantly lower likelihood of good practices towards vitamin D (cOR = 0.41, 95% CI: (0.25–0.68); *p* = 0.001). Married individuals showed an increased likelihood of good practices compared to widow(er)s (cOR = 2.84, 95% CI: (1.17–6.92); *p* = 0.022). Basic education (cOR = 4.97, 95% CI: (2.99–8.26); *p* < 0.001) and SHS education (cOR = 5.80, 95% CI: (3.32–10.13); *p* < 0.001) increased the likelihood of good practices compared to tertiary education. Students had a decreased likelihood of good practices compared to unemployed individuals (cOR = 0.31, 95% CI: (0.12–0.79); *p* = 0.014). Speaking Twi increased the likelihood of good practices (cOR = 2.84, 95% CI: (1.04–7.72); *p* = 0.041). Being overweight also increased the likelihood of good practices (cOR = 3.18, 95% CI: (1.23–8.23); *p* = 0.017).

However, in the multivariate logistic regression analysis, having basic education (aOR = 9.06, 95% CI: (2.45–33.51); *p* = 0.001) and SHS education (aOR = 5.25, 95% CI: (2.51–11.00); *p* < 0.001) were associated with increased odds of good practices regarding vitamin D (Table 8).

**Table 8:** Sociodemographic and lifestyle predictors of good practice towards vitamin D

Variable	Good Practice (*n* = 377)	cOR (95% CI)	*p*-value	aOR (95% CI)	*p*-value
**Age (yrs)**					
18–24	148 (39.3)	0.41 (0.25–0.68)	**0.001**	1.60 (0.53–4.82)	0.401
25–39	121 (32.1)	1.17 (0.64–2.14)	0.622	-	-
≥ 40	108 (28.6)	1		1	
**Marital Status**					
Single	192 (50.9)	0.82 (0.36–1.85)	0.623	-	-
Married	159 (42.2)	2.84 (1.17–6.92)	**0.022**	1.89 (0.63–5.70)	0.260
Divorced	5 (1.3)	0.54 (0.12–2.47)	0.424	-	-
Widow(er)	21 (5.6)	1		1	
**Highest Educational Level**					
Non-formal Education	1 (0.3)	0.43 (0.04–4.86)	0.498	-	-
Basic Education	143 (37.9)	4.97 (2.99–8.26)	** < 0.001**	9.06 (2.45–33.51)	**0.001**
SHS	127 (33.7)	5.80 (3.32–10.13)	** < 0.001**	5.25 (2.51–11.00)	** < 0.001**
Tertiary	106 (28.1)	1		1	
**Employment status**					
Unemployed	26 (6.9)	1		1	
Student	129 (34.2)	0.31 (0.12–0.79)	**0.014**	0.32 (0.09–1.17)	0.085
Self-employed	201 (53.3)	1.41 (0.54–3.68)	0.487	-	-
Formal	16 (4.2)	0.92 (0.23–3.78)	0.911	-	-
Retired	5 (1.3)	-	0.999	-	-
**Language**					
Twi	369 (97.9)	2.84 (1.04–7.72)	**0.041**	1.01 (0.35–2.46)	0.991
No	8 (2.1)	1		1	
**English**					
Yes	222 (58.9)	0.45 (0.29–0.70)	** < 0.001**	2.36 (0.84–6.64)	0.104
No	155 (41.1)	1		1	
**Others**					
Yes	58 (15.7)	0.60 (0.37–0.98)	**0.040**	1.02 (0.58–1.78)	0.941
No	319 (84.6)	1		1	
**BMI**					
Normal	330 (87.5)	1		1	
Underweight	6 (1.6)	0.58 (0.16–2.10)	0.407	-	-
Overweight	41 (10.9)	3.18 (1.23–8.23)	**0.017**	2.23 (0.78–6.38)	0.135
Obese	0 (0.0)	-	-	-	-

*cOR* crude odds ratio, *aOR* adjusted odds ratio, *CI* Confidence interval, *p* < 0.05 and bolded means statistically significant

## Discussion

Despite Ghana's abundant sunshine, vitamin D deficiency is prevalent among the adult population [1, 11, 12]. Studies from other parts of the world have linked vitamin D deficiency to lack of awareness and knowledge regarding vitamin D [19, 20]. Therefore, we investigated vitamin D awareness, knowledge, and associated factors in the Jaman South District, Ghana. The study revealed varying levels of awareness, knowledge, attitude, and practices related to vitamin D among the participants. Sociodemographic factors, including age, education, employment status, and language proficiency, were found to significantly influence individuals' knowledge and practices regarding vitamin D.

This study found that 61% of participants were aware of Vitamin D, a significantly lower rate compared to a previous study in Malaysia, which reported an awareness level of 90.5% [21]. Similarly, previous studies from Egypt (94.3%) and Saudi Arabia (89.6%) reported higher levels of awareness compared to the current study [22, 23]. In Ghana, public health education campaigns primarily focus on chronic diseases such as diabetes and hypertension, which often leads to reduced attention on nutrient deficiencies like Vitamin D deficiency.

Over 56% of the study participants demonstrated adequate knowledge of Vitamin D. However, a substantial number (42.7%) were unaware of its dietary sources, which is considerably higher than the findings reported in Malaysia and India [24, 25]. The study found that knowledge of vitamin D sources was primarily limited to sun exposure (53%) and diet (42.7%). This is consistent with the findings of Mithal et al., (2009) [26] and also a French study, which reported that while many are aware of sun exposure as a source of vitamin D, fewer people are aware of dietary sources [27]. Similar to findings from a previous study in the US, 86.6% of respondents in our study recognized outdoor activities as beneficial for sun exposure [28].

This linguistic factor may reflect broader access to English-language health information and resources, highlighting potential disparities in health communication. There is therefore a need for Vitamin D related information to be translated to local dialects to facilitate broader coverage. A study by Babelghaith and colleagues similarly found that English proficiency was associated with better vitamin D knowledge among university students [29].

A significant proportion of participants (44.5%) reported that they do not expose themselves to sunlight all the time. A study in Malaysia found that only 17% of participants do not regularly expose themselves to sunlight [21]. Our study also showed that being male was associated with over 3 times increased likelihood of having a satisfactory attitude compared to females. This observation conflicts with the findings of a study conducted by Boland et al., (2015) in Ireland found that women had significantly higher vitamin D knowledge scores compared to men [16].

Furthermore, we found that only 5.2% of participants reported taking vitamin D supplements, which is significantly lower than rates observed in countries like Bahrain where over 50% of adults reported using Vitamin D supplement [30]. This low rate of supplement use in Jaman South could be due to the low awareness regarding Vitamin D as highlighted in this study. Contrary to previous studies which found an association between higher education and good practice regarding Vitamin D [16, 31], our study reported an association between lower levels of education and better vitamin D practices. One possible reason for this disparity is that there might be more effective vitamin D education programs targeted at basic and secondary education levels in the studied population.

Several factors were associated with vitamin D practices, including age, marital status, employment status, language proficiency, and BMI. Younger individuals (18–24 years) were less likely to have good practices compared to those 40 and above, which aligns with findings from Hong Kong, where older adults demonstrated better vitamin D knowledge and practices [32]. Married individuals showed better practices compared to widowed individuals, possibly due to mutual health support within marriages, a trend also observed in Australia [31]. The association between Twi (local) language proficiency and good practices shows the importance of culturally appropriate health education, as emphasized in research by Alemu and Varnam, (2012) in England [33]. Interestingly, being overweight was associated with better practices which contrasts with some studies linking higher BMI to lower vitamin D levels, such as the research by Vimaleswaran et al., (2013) [34]. This could indicate increased health awareness and preventive practices among overweight individuals in the studied population.

By building on the findings from this study, public health strategies can be refined to effectively promote good vitamin D practices across all segments of the population, ultimately contributing to better overall health outcomes. Although this study offers valuable insights into the factors influencing knowledge, attitudes, and practices regarding Vitamin D, it is limited by its focus on a rural setting predominantly comprising individuals with lower income levels. This demographic profile may have influenced the study outcomes and limits the generalizability of the findings to more diverse or urban populations.

## Conclusion

There is high awareness but reduced knowledge on Vitamin D among the general public in Jaman South. The majority of participants had a satisfactory attitude about the attainment of vitamin D with very high practice levels. Age, education, employment status, language (English) were the factors significantly associated with knowledge and practice regarding vitamin D. Therefore, future research should focus on designing and evaluating extensive health education campaigns aimed at improving public knowledge on the importance of vitamin D and promoting practices that enhance vitamin D sufficiency.

## Data Availability

The datasets used and/or analyzed during this study are available from the corresponding author on request.
